# Recalcitrant Esophageal Stricture Secondary to Mycophenolate Mofetil

**DOI:** 10.1155/2020/8817801

**Published:** 2020-11-23

**Authors:** Joyce J. Kim, Ramzi H. Mulki, Kavya M. Sebastian

**Affiliations:** ^1^Department of Medicine, Emory University School of Medicine, Atlanta, Georgia; ^2^Department of Medicine, Division of Digestive Diseases, Emory University School of Medicine, Atlanta, Georgia

## Abstract

Mycophenolate mofetil (MMF) is associated with various gastrointestinal toxicities. However, limited literature studies exist reporting MMF-related gastrointestinal toxicity manifesting as esophageal strictures. We report a case of a 62-year-old male with kidney transplant on MMF, tacrolimus, and prednisone, presenting with progressive dysphagia and odynophagia. Esophagogastroduodenoscopy revealed severe esophageal stricturing with near food bolus impaction, requiring dilations, esophageal stent, and ultimately gastrostomy tube. Biopsies revealed nonspecific inflammation with no evidence of infectious/neoplastic process; thus, our multidisciplinary esophageal group determined that the process was secondary to MMF. This case demonstrates that, though rare, MMF can result in severe esophageal strictures causing significant morbidity.

## 1. Introduction

Advances in immunosuppressive agents have transformed the field of transplantation, improving graft and patient outcomes [1, 2]. Unfortunately, these medications also commonly lead to gastrointestinal (GI) complications. Specifically, mycophenolate mofetil (MMF) has been associated with increased risk of infectious esophagitis [3], GI malignancies [4], as well as injury to both upper and lower gastrointestinal mucosa manifesting as constellation of symptoms including nausea, vomiting, abdominal discomfort, and diarrhea [5–8]. However, limited literature exists on esophageal stricture as a complication of MMF. Herein, we present a rare case of recalcitrant esophageal stricture caused by MMF.

## 2. Case Report

**Figure 1:**
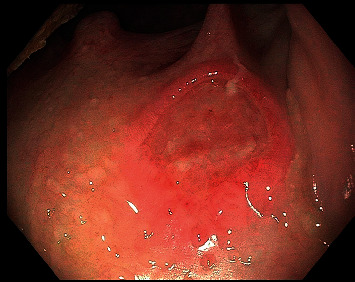
Soft palate ulcer in the right oropharynx, which was biopsied and revealed nonspecific findings of acute and chronic inflammation.

**Figure 2:**
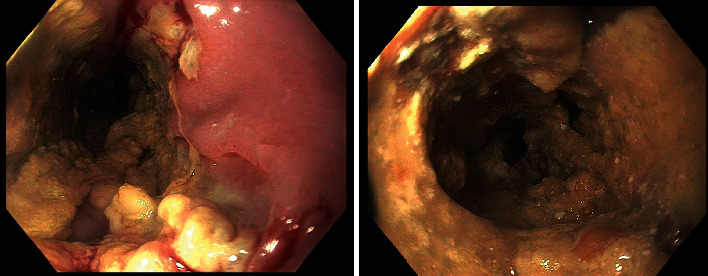
Multiple, deeply cratered ulcers, in a circumferential manner with adherent food debris (a), causing severe narrowing of the esophageal lumen, in a near complete food bolus impaction (b).

**Figure 3:**
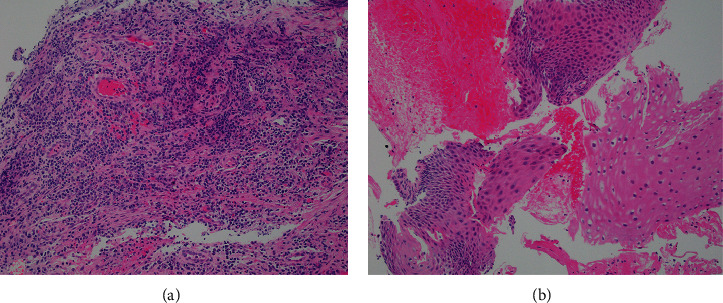
Nonspecific histologic findings included granulation tissue and necroinflammatory debris (a) consistent with endoscopically identified ulcer. Fragments of squamous epithelium showed reactive features (basal cell hyperplasia and balloon cell change) (b).

We report a case of a 62-year-old male with a history of renal transplant (5 months prior to presentation) on an immunosuppressive regimen consisting of MMF, tacrolimus, and prednisone, who presented with progressive solid and liquid dysphagia and odynophagia for one month, with associated 20-pound weight loss. An EGD was performed, and upon careful inspection, a shallow 1  cm × 1  cm ulcer was seen on the right side of the soft palate (Figure 1). With further advancement, multiple, deeply cratered ulcers, in a circumferential manner, were found starting 23 cm from the incisors to the GE junction (40 cm from the incisors) (Figure 2(a)). Food debris were adherent to the ulcers, causing severe narrowing of the esophageal lumen in a near bolus food impaction, and was only traversable with an ultrathin endoscope (Figure 2(b)). Biopsies were obtained from the center and the edge of the ulcers, which revealed reactive squamous mucosa with fragments of granulation tissue and neuroinflammatory debris (Figure 3). Immunostains for CMV, HSV1/2, and fungal organisms were negative. Otolaryngology obtained a biopsy of the ulcerative lesion found in the soft palate during initial EGD, which revealed nonspecific findings of acute and chronic inflammation and negative for infectious causes.

**Figure 4:**
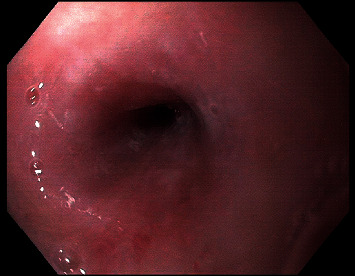
EGD revealing a pin-point esophageal in the midesophagus.

**Figure 5:**
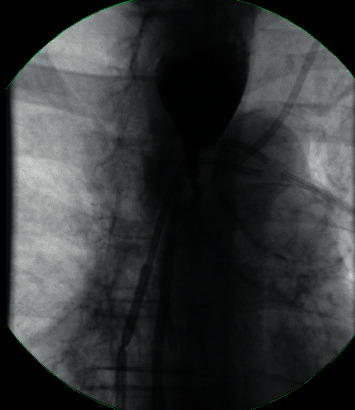
Barium swallow demonstrating a severe narrowing in the mid and distal esophagus with proximal dilation.

**Figure 6:**
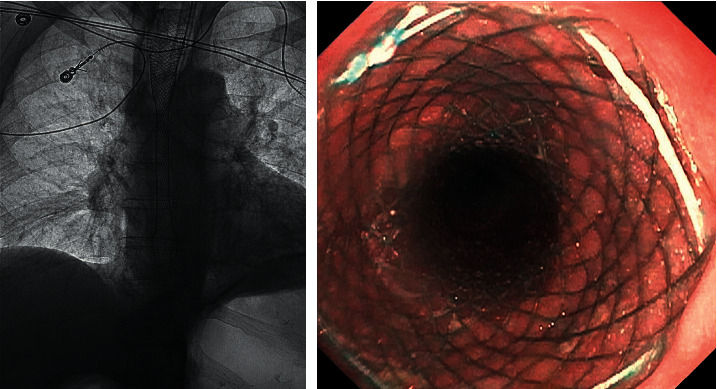
A fully covered esophageal metal stent placement under fluoroscopy.

The patient was discharged on high-dose proton pump inhibitor, liquid diet, and total parenteral nutrition. MMF was suspended given the suspicion of MMF-induced esophageal injury. A repeat EGD 4 weeks later revealed a pin-point esophageal lumen starting from 23 cm from incisors not traversable with the ultrathin upper endoscope (Figure 4). A barium swallow demonstrated the extent of the narrowing in the mid and distal esophagus with proximal esophageal dilation (Figure 5). The patient was referred for a surgically placed feeding gastrostomy tube, with plans for serial dilation under fluoroscopic guidance. Several attempts at endoscopic dilation were performed using a through-the-scope (TTS) balloon dilator from 6 mm to 10 mm. The esophageal stricture was recalcitrant despite dilation and maximal medical therapy. After discussion in multidisciplinary dysphagia conference, the decision was made to proceed with esophageal stenting. This was performed using a 18  mm × 12.3  cm fully covered stent, which was placed under fluoroscopic guidance (Figure 6). The patient tolerated the procedure initially, but due to chest discomfort, the stent was subsequently removed. Esophagectomy was considered but deferred due to the patient's comorbid conditions; he opted for maximal medical therapy (fluticasone 2 puffs of 220 mcg/inhalation aerosol twice daily, lansoprazole 30 mg twice daily, and sucralfate 1 g/10 mL solution 4 times daily) and serial dilations with intralesional steroid injections. A year and a half after initial presentation, his oral intake is improving with slow weaning off of dependence on feeding tube.

## 3. Discussion

MMF is an immunosuppressive agent that selectively acts on T and B lymphocytes and is commonly used to prevent graft rejection after transplantation [9]. Unfortunately, MMF has been associated with gastrointestinal toxicities with reported incidence varying from 40% to 85% [10]. Previous studies have suggested that mucosal injuries throughout gastrointestinal tract can occur particularly within the first twelve months of initiating MMF; one study found that, among those with kidney transplants on immunosuppressants, 46% developed ulcers in the first year, with MMF being an independent risk factor [11].

Though not fully understood, MMF-related complications in the GI tract have been attributed to its mechanism of inhibiting lymphocyte proliferation by blocking de novo guanosine nucleotide synthesis, upon which the rapidly replicating enterocytes partially depend, thereby disrupting the gastrointestinal epithelial barrier [12]. In addition, its metabolites including mycophenolic acid acyl-glucuronide (AcMPAG) can trigger the immune system, leading to hypersensitivity and autoimmune-like reactions [5]. Interestingly, the reported pathologic features of MMF-related toxicity of upper and lower GI tract are dissimilar. Most extensively documented are graft-versus-host disease such as changes of the colon and small intestines including mild crypt disarray, crypt loss, and increased epithelial apoptosis (features that are nonspecific on their own) [7, 8, 13], symptomatically manifesting as diarrhea, and a presentation similar to inflammatory bowel disease. In contrast, upper GI toxicities of MMF have been found to be similar to features of nonsteroidal anti-inflammatory drug use with topical irritation and damage, leading to ulcerative esophagitis, reactive gastropathy, and duodenal ulcers [7, 14, 15]. This can symptomatically manifest as nausea/vomiting, dysphagia, and dyspepsia.

However, to our knowledge, manifestation of MMF-related upper GI toxicity as esophageal stricture is not well-reported. This case demonstrated an example of a recalcitrant esophageal stricture causing near food bolus impaction, requiring multiple dilations, esophageal stent placement, and ultimately gastrostomy placement. Given multiple biopsies of nonspecific findings (reactive squamous mucosa) with negative stains for infectious or neoplastic processes, the disease process was attributed to MMF. One of these biopsies included an ulcerative lesion of the oral cavity that was found during the initial EGD; an oropharyngeal examination is often overlooked during a routine EGD but may reveal incidental findings with important implications [16]. The recurring nature of the stricture in this case suggests that there likely was an underlying fibrotic process that occurred in an irreversible manner.

This report contributes to a better understanding of atypical presentation of MMF-induced esophagitis in immunocompromised patients, expanding upon prior reports regarding upper GI manifestation of MMF. Specifically, this report demonstrated a new potential consequence of MMF in the esophagus, namely, strictures. This diagnosis should be considered in a posttransplant patient with dysphagia when more common etiologies (e.g., reflux esophagitis or infectious esophagitis) have been excluded. Furthermore, an improved systematic surveillance may be needed among patients on immunosuppressants, particularly MMF, given that delay in appropriate diagnosis and treatment can result in significant morbidity, as well as possible life-threatening consequences, as in our case.
